# Heterogeneity among Venezuelan migrants in terms of coping in the context of the population exodus from Venezuela

**DOI:** 10.1371/journal.pone.0332084

**Published:** 2025-09-15

**Authors:** Marcin Stonawski

**Affiliations:** 1 Center for Advanced Studies of Population and Religion (CASPAR), Krakow University of Economics, Krakow, Poland; 2 International Institute for Applied Systems Analysis (IIASA), Laxenburg, Austria; Fundación Universitaria del Área Andina, COLOMBIA

## Abstract

This study concentrates on the unparalleled exodus of Venezuelans in recent years. By June 2024, the global population of Venezuelan migrants and refugees had reached 7.7 million, with 6.6 million settling in Latin America. This represents one of the most significant migration outflows of the 21st century, with estimated numbers exceeding those of emigration from Afghanistan, Ukraine and Syria. However, coping strategies and integration of Venezuelans in hosting countries is understudied phenomenon in comparison to other migration movements. The aim of this paper is to examine patterns of coping among Venezuelan migrants. By analyzing coping strategies, we aim to contribute to enhance understanding of the characteristics of Venezuelan migrants in the context of their adaptation in the host countries in Latin America. We conducted a survey among Venezuelan migrants in Peru in 2023. The study is based on the Lazarus and Folkman model of stress and coping. A Coping Strategy Inventory-SF instrument and a Latent Class Analysis method were employed to distinguish three homogeneous subgroups in terms of coping strategies. We found three such subgroups: problem engagers, hybrid engagers and mixed strategy users. These groups exhibit distinct characteristics with regards to age, sex, education and optimism. The article highlights the heterogeneity in the use of coping strategies among Venezuelan migrants. To the best of our knowledge, this study is the first one to apply Lazarus and Folkman’s model in conjunction with Latent Class Analysis in the field of migration studies. We strongly believe the proposed approach is useful in increasing our understanding about coping strategies and integration among migrant populations.

## Introduction

The Venezuelan exodus driven by economic deterioration, collapsing health system, violence and political instability in their home country has significant impact on the migration system in the whole of Latin America and implication for the global migration patterns [1–3]. The country, which had been a major recipient of migrants in Latin America for decades, became in a very short time the largest sender not only in Latin America but also in the world. By June 2024, the global population of Venezuelan migrants and refugees had reached 7.7 million, with 6.6 million establishing residence in Latin America and the Caribbean according to 2024 estimates of Regional Inter-Agency Coordination Platform for Refugees and Migrants from Venezuela (R4V). This represents one of the most significant migration outflows of the 21^st^ century, with estimated numbers exceeding those of emigration from Afghanistan, Ukraine and Syria. As previously stated, Venezuelans have no emigration experience that could accumulate in the society and prepare them to cope with challenges of migration process that have experience in the recent decade. Consequently, it is important to gather more knowledge about utilization of coping strategies among Venezuelan migrants. Better understanding of individual behaviors in stressful situations within the aforementioned group could prove to be valuable in formulating thoughtful integration policies by the host countries. Our article aims to fill this knowledge gap providing examination of coping strategies of Venezuelans residing in Peru, which has become one of the principal destinations for Venezuelans with a migrant population of 1.5 million. Furthermore, this article proposes a typology of Venezuelan migrants in this context. This study is exploratory in nature, as, to the best of our knowledge, no previous studies on migration have attempted to analyze the general adult migrant population in terms of coping strategies despite the vast number of publications that have focused on specific subgroups of migrants in terms of demographic, socio-economic characteristics etc. We apply the instrument predominantly used in health studies, including migrant health – Coping Strategy Inventory, and combine it with Latent Class Analysis to distinguish homogeneous migrant groups related to their self-declared behaviors in stressful situations. We argue that this approach can be useful in better understanding immigrants’ populations and their adaptive capabilities around the world.

### Literature review

Coping can be defined as the behaviors and cognitive skills that individuals utilize in order to cope with internal and environmental demands that are perceived as stressful [4]. The level of stress is heightened when the individuals are confronted with significant life challenges [5]. One such challenge is migration, which creates a profound change in life and challenges for the individual. The decision to migrate and its subsequent implementation is a consequence of push factors present in the country of origin which become stressors for the individuals and their families (e.g., lack of employment and income, low security, hunger, lack of access to health services, education). In this context, migration can be considered an answer and a coping strategy in response to the situation [6]. However, the migratory experience on the individual level is accompanied with a significant level of stress at each step of the process [7–10]. Studies of literature provide summary of challenges that migrants face during their migration process [11,12]. Consequently, migration can be considered a coping strategy that creates additional stress, which the migrant must then manage [13]. This has a significant impact on mental and physical health of immigrants [14–17]. Yakushko et al. [12] identify a number of stressors associated with the migration process: (a) pre-migration stressors, which are associated with previous experiences from an origin area and include conflict, violence, trauma, fear associated with unknown future, and (b) post-migration stressors, which are related to changing place of residence, acculturative stress, health problems, loss of social status and contact with the family and already-built social networks, and oppression by the host society (e.g., discrimination). Berry [18] introduces the concept of acculturation stress which refers to the stress experienced by immigrants as a result of their interaction with the host society. The ability to cope is of paramount importance for migrants in order to successfully navigate the challenges associated with integration and to facilitate their adaptation to the new culture [19]. Upon arrival, immigrants are compelled to determine and execute coping strategies in order to subsist, survive and flourish in an alien environment. The negative consequences of the applied coping strategies may result in social and economic marginalization of an individual or family unit that is isolated from both the local population and other immigrants, or separation from hosting society. In the most extreme cases, this can result in a situation where the individual or family lacks the resources to return to their country of origin [20]. A positive outcome of the aforementioned tactics enables the immigrant to remain in the destination country, applying an assimilation or integration strategy, following Berry’s model [18]. Moreover, the migrant can also return to the country of destination or continue migration to another country.

In their seminal work, Lazarus and Folkman [4] identify two distinct coping strategies: (a) problem-focused which attempts to change or solve the problem, and (b) emotion-focused, which aims to regulate emotions associated with the problem. Furthermore, Tobin et al. [21] propose a second-order division to Lazarus and Folkman’s [4] typology, distinguishing engagement coping strategies, which are active endeavors to manage stressful situations (e.g., problem-solving, seeking social support), and disengagement coping strategies, which are related to behaviors and thoughts that evade the modification of the situation (e.g., self-criticism, wishful thinking). Individuals may employ a combination of coping strategies in response to stressful circumstances. There is no single effective coping strategy because one strategy can be effective in a specific situation but not necessarily in other situation [22,11]. However, individuals may be inclined to utilize a particular combination of these strategies, with a tendency towards one or other dimension (emotional versus problem-focused, engagement versus disengagement). Lazarus and Folkman [4] suggest that individuals who are exposed to a similar stressor may experience and exhibit disparate physical and psychological reactions due to their individual predispositions. Empirical evidence indicates that there is also a cross-cultural variation in the preferences of coping strategies [23–27].

In the case of Latin American migrants and their descendants, studies from the United States indicate that they more often use support from family and their social network than other non-Latino groups, for example Black population [9,28,29]. A substantial body of evidence suggests that Latin American-origin migrants of various generations possess extensive supportive social networks that help them in dealing with stress [29–32]. Okumura et al. [33] provide evidence on positive impact of social support (family, friends, and significant others) on health outcomes among Venezuelan migrants in Peru. This phenomenon is closely linked to familism, a cultural value that emphasizes the obligation of family members to provide emotional and instrumental social support when needed [34]. Familism is widely regarded as a core tenet of Latino culture [35–37]. An additional significant coping strategy for Latin Americans is religiosity, which facilitates the management of stress in Latino migrants [38,39]. This coping style is particularly prevalent among less acculturated migrants in the US [40]. In religious coping, we can find emotion-focused engagement strategies, when an individual for example, consult priest or religious community, attend church, or participate in other religious activities [41]. For example, the 2018 ISSP survey indicates high level of religiosity in Venezuela, with 60% of respondents reporting that they prayed at least two or three times a month (31% several times a week) and approximately 40% attending church at least two to three times a month [42]. However, the religious coping can be associated with disengagement strategies, such as fatalistic attitudes (belief in predetermined fate, seeking a miracle, etc.). These attitudes were of particular significance in Venezuela. For example, approximately 80% of the population believed in religious miracles, and nearly 30% indicated that people can do little to change the course/destiny of their lives [42]. Constantine et al. [43] find a significant prevalence of disengagement strategies, including denial, among Latinos in the United States. Therefore, we hypothesize that Venezuelan migrants frequently employ, together with problem-focused engagement strategies, emotional engagement and disengagement strategies.

## Materials and methods

In our analysis, we utilise data from our own survey of 305 Venezuelans aged 18 and over, residing in Peru. This survey was conducted as part of a research project, entitled “*Migration Crisis in Latin America – coping and adaptation strategies of Venezuelan migrants and their families and the risk of global migration crisis [MICLACAS]*”. The fieldwork was conducted between 25 April and 15 May 2023 using the face-to-face computer-assisted personal interview (CAPI) method. It is estimated that between 75 and 82 percent of Venezuelans residing in Peru live in an agglomeration around Lima, the capital city [44,45]. Consequently, our sampling was confined to this geographical area, in consideration of the a priori knowledge available regarding the spatial distribution of Venezuelans in Lima and Callao (see: INEI studies including [46,47]), as well as the objectives and costs of the study. The survey was conducted in all districts within the city of Lima, including Lima Norte, Lima Este, Lima Sur, Lima Centro, Lima Moderna, and Callao. The sample included respondents from 37 districts within this area. The selection criteria for the sample were Venezuelan nationality and principal residence in Peru, as well as staying in a particular location five to seven days a week (in order to eliminate the possibility of guests or tourists, etc.), regardless of the time of arrival or legal status in Peru. However, before 2015, migration of Venezuelans to Peru was negligible – according to UN estimates, there were only around 26 thousand Venezuelans in Peru in 2014, compared to 1.6 million in 2024 (see detailed demographic analysis of migration from Venezuela in [2]). This is reflected in the sample: only four respondents arrived in Peru before 2015, while around 62% arrived during the peak of the exodus in 2017–2019.

Due to the lack of a comprehensive registry of Venezuelan migrants in Peru, many of whom reside in an irregular status, obtaining a fully statistically representative sample for Peru or Lima was not feasible. Instead, the sampling strategy was based on the ENPOVE-2022 survey, the most reliable data source available. The procedure was implemented with the objective of achieving a representative sample in the context of the aforementioned information deficit. We employed a random spatial sampling method in grid areas (100m x 100m) with high estimated concentrations of Venezuelan migrants, as identified in the ENPOVE study. Initially, 300 grids were selected with plan to conduct one efective survey in the area. If no eligible individuals were found in a selected grid, the next grid was used. In cases of 8 or more Venezuelan families resinding at one property, interviewers could conduct interviews with representatives of two families. The inclusion criteria followed ENPOVE-based quotas for age, sex, education, and district, ensuring alignment with known population distributions. Given this alignment, no additional weights were applied.

However, this strategy may under-represent migrants in lower-density areas, who are potentially more integrated into Peruvian society. Consequently, the findings are likely to be conservative with regard to issues associated with integration, including the level of utilisation of some coping strategies.

The fieldwork survey was commissioned by our research team to Datum International, a well-recognised survey company in Peru and it was conducted in accordance with the ISO norm 20252. The study was approved by the University Ethics Committee for Scientific Research at the Krakow University of Economics, in accordance with ethical principles, data protection regulations and good practice in scientific research [decision no. KEBN/71/0044/D18/2023]. All participants agreed to take part in the survey and gave verbal informed consent after an interviewer had carefully read the text. All consents were audio-recorded by DATUM staff.

**Fig 1:**
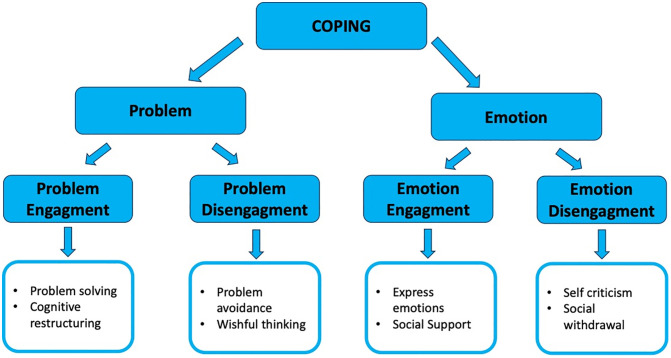
The hierarchical structure of coping Source: own graphics based on Tobin et al. (1989).

In order to evaluate the coping strategies employed by Venezuelan migrants in Peru, we utilize the instrument developed by Addison et al. [48] called the Coping Strategy Inventory – Short Form (CSI-SF). This is a condensed version of the 72-item CSI scale created by Tobin et al. [21] which was based on a seminal work of Folkman and Lazarus [4] on the Ways of Coping Questionnaire (WCQ). The CSI-SF comprises of 16 items and exhibits a comparable structure to that the original CSI scale. The instrument includes items from all four subscales, namely (a) *Problem–Focused Engagement*; (b) *Problem‐Focused Disengagement*; (c) *Emotion‐Focused Engagement*, and (d) *Emotion‐Focused Disengagement* (Fig 1). The responses are recorded on a five-point Likert scale, which reflects the frequency with which respondents employ each coping strategy enumerated in the survey. Responses vary from never, rarely, sometimes, almost always, to always. The total score for a given subscale is calculated by summing the responses to all items within that subscale. In conducting a survey of Spanish-speaking respondents in the Latin American context, we utilize a Spanish version of the instrument that has been developed and tested by Tous-Pallarés [49]. The descriptive findings of the paper present population as divided into three categories in each of the subscales: low (4–9 points), medium (10–15), and high (16–20) (overall alpha-Cronbach coefficient = 0.63). The cutoffs have been selected based on the range of scores of the instrument, which is 4–20 points for each type of coping strategy. The three groups were derived by dividing the range into three equal segments based on the score.

To classify Venezuelan migrants into distinct subgroups based on their response pattern to questions of CSI-SF instrument, the latent class analysis (LCA) was applied within the framework of generalized structural equation model (GSEM) using STATA software (*gsem* command). LCA uses a person-centered approach instead of a variable-centered approach (Factor Analysis). The goal of this method is a distinct and parsimonious classification of individuals into latent subgroups by maximizing heterogeneity between and homogeneity within subgroups [50,51]. For the analysis responses for questions in CSI-SF instrument were collapsed into 1 if use of a given strategy was at least often (often, almost always), and 0 if it was sometimes or lower (never, rarely, sometimes). We use modal assignment to the groups – each individual is assigned to the class for their posterior probability is the highest. This approach does not account for classification uncertainty. However, the estimated entropy is on the level of 0.93, that suggests good class separation. Thus, bias should be tolerable to describe characteristics of the subgroups.

Furthermore, to identify distinctive demographic and socio-economic characteristics of persons within each subgroup we use the logit regression models. A distinct model was constructed for each subgroup, with the objective of determining the probability of belonging to a particular group. In the analysis, the following variables were included: age, sex, education level divided into three categories: primary and lower (ISCED 0–1), secondary (ISCED 2) and postsecondary and higher (ISCED 5+), and migration intentions (describing whether a person plans to stay permanently in Peru or wants to migrate. Additionally, the measure of optimism was incorporated into the analysis as potentially important variable associated with coping mechanism [52]. We measure optimism using a well-accepted instrument proposed by Scheier et al. [53] called Revised Life Orientation Test (LOT-R). It comprises 10 items, which are divided into two subscales: optimism and pessimism. A five-level Likert scale was employed to score each question, with a total range of 0–4 points. In the study, we utilize a Spanish version of the test proposed and validated by Otero-Lopez et al. [54], a test extensively employed in Spanish-speaking countries [55,56]. The respondents were classified into three categories based on their scores, which were as follows: pessimism (score 0–13), moderate optimism (14/18) and high optimism (19/24). The cutoffs divide populations roughly 25% − 50% − 25%.

## Results and discussion

### Descriptive findings

The Venezuelan migrant sample utilized in the present study encompasses 305 individuals. The demographic profile of the dominant group shows that the majority of its members are young adults, with 44% of the sample falling into the 18–29 age category and 35% within the 30–44 age bracket (see Table 1). The proportion of individuals aged 45 and over is 21%. The population is characterized by a balanced ratio of males to females, with 51% of the population comprising men and 49% comprising women. With regard to the self-reported educational attainment of the participants, the majority (51%) reported having completed secondary education, 36% reported having completed post-secondary education, and 13% reported having primary or lower level of education. Approximately 37% of respondents expressed intentions to reside permanently in Peru, while 63% indicated a desire to depart from Peru with the intention of returning to Venezuela (approximately 40%) or continuing their migration to other countries (23%). In relation to optimism, the mean LOT-R score was 16 points (SD 3.7). The data revealed that approximately 23% of participants obtained a score below 14 points, 51% attained a score between 14 and 18 points, and 26% achieved a score above 18 points.

**Table 1:** Chosen characteristics of Venezuelan migrants in the study.

Variable	*Sample composition*
**Age**	
18-29	44%
30-44	35%
45+	21%
**Sex**	
Males	51%
Females	49%
**Education**	
Primary and lower	13%
Secondary	51%
Postsecondary+	36%
**Optimism scale**	
Pesimists	24%
Moderate Optimism	51%
High Optimism	25%
**Migraton Intentions**	
Leave Peru	63%
Stay in Peru	37%
***Sample size***	**305**

Source: own calculations based on the 2023 MICLACAS survey.

**Fig 2:**
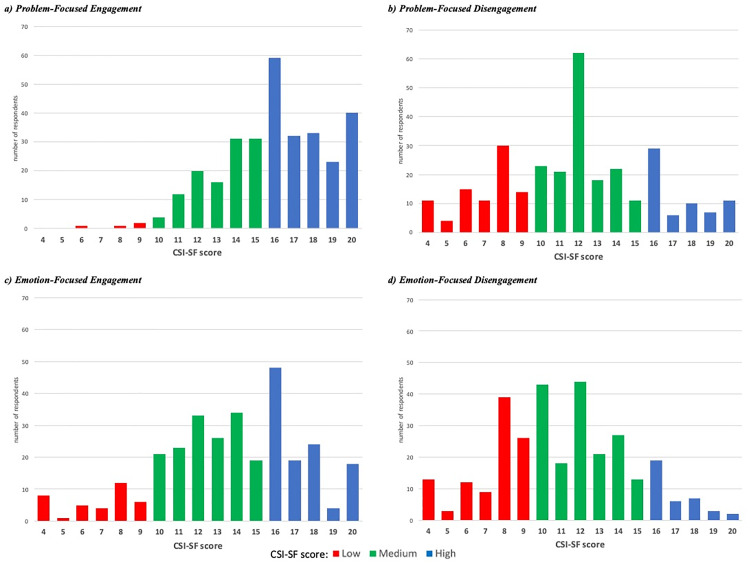
Distribution of CSI-SF scale: Source: own calculations based on the 2023 MICLACAS survey.

**Fig 3:**
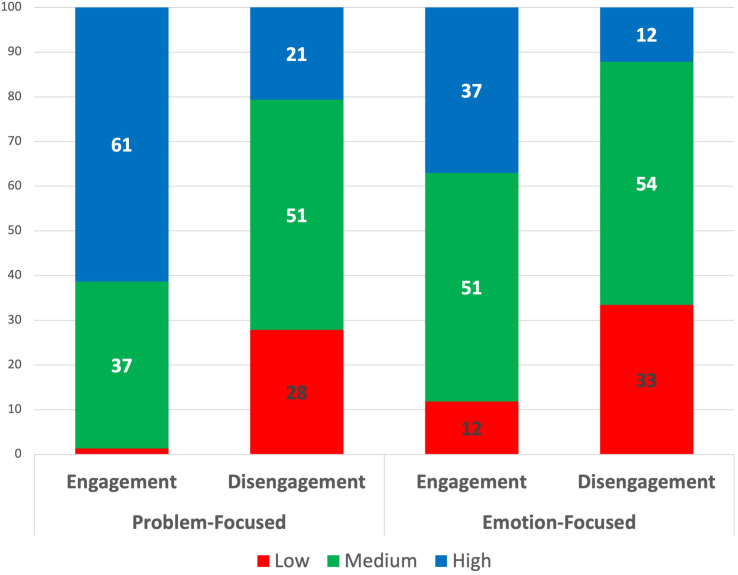
Distribution of Venezuelan migrants by CSI-SF subgroups. Source: own calculations based on the 2023 MICLACAS survey.

The findings provide evidence that Venezuelan migrants tend to employ engagement coping strategies with greater frequency, as illustrated in Fig 2. This is particularly evident in the case of problem-focused engagement strategies, as illustrated in the distribution on Fig 2a, which demonstrates a high frequency of scores above 15 points. A total of 61% of respondents exhibited high scores, indicating a proclivity for problem-focused engagement strategies (e.g., planning, problem-solving) (see Fig 3).

It appears that problem-focused disengagement strategies are not a prevalent phenomenon among migrants. Fig 2b demonstrates that the majority of migrants exhibit low and medium scores on the scale. Approximately 28% of the sample is situated in the low group and only 21% in the high group, indicating that they engage in this kind of behavior with a high degree of frequency (e.g., allowing the problem to be resolved by itself, avoiding thinking about the problem or hoping for a miracle).

In the case of stress, Venezuelan migrants in Peru frequently employ emotion-focused engagement coping strategies, such as discussing their feelings with friends and family or expressing their emotions to reduce stress. Approximately 37% of respondents exhibited high scores on the scale, with the majority situated at the midpoint of the distribution (Fig 2c). Only 12% of respondents indicated that they seldom or never utilize these strategies.

A notable proportion of Venezuelans refrain from emotion-focused disengagement strategies, including activities such as spending time alone, self-criticism, and the isolation of emotions and thoughts (Fig 2d). One-third of the migrants surveyed did not employ these strategies or did so on an occasional basis, resulting in a low score on the scale.

Table 2 presents the average scores for each subcategory of coping. Venezuelan migrants residing in Peru get the highest score in *problem-focused engagement* strategies – 16 out of 20 points – with corresponds to high level. The next are emotion-focused engagement strategies, which have an average score of 13.6 points. The lowest scores were observed for disengagement strategies, both problem-focused and emotion-focused, with an average of 11.8 and 11.1 points, respectively.

**Table 2:** Descriptive statistics of CSI-SF score subscales.

Indicator		Mean	SD
Problem-Focused	Engagement	15.97	2.77
	Disengagement	11.83	3.91
Emotion-Focused	Engagement	13.65	3.70
	Disengagement	11.08	3.47

Source: own calculations based on the 2023 MICLACAS survey.

### Latent class analysis

Our findings revealed significant discrepancies in the utilization of coping strategies among Venezuelan migrants, contingent on their responses to the CSI-SF instrument questions. We conducted latent class modelling at varying levels of classification and evaluated the goodness-of-fit statistics. The analysis indicates that a three-class model is the most appropriate for our data in terms of parsimony, goodness of fit, and interpretability (see Table 3).

**Table 3:** Summary of Information for Selecting Number of Latent Classes.

No. of classes	Likelihood ratio G	Degree of freedom	AIC	BIC
1	−2928.835	16	5889.669	5949.194
2	−2801.837	33	5669.674	5792.444
**3**	**−2761.266**	**50**	**5622.532**	**5808.548**
4^*^	−2734.111	66	5600.221	5845.762
5^*^	−2715.153	78	5586.307	5876.491

Source: own calculations based on the 2023 MICLACAS survey.

Note:

*the convergence was not achieved in the baseline GSEM model, the table includes results from the restricted models for 4 and 5 classes.

In light of the aforementioned evidence, we proceeded to calculate the probabilities of belonging to each class for the three-class model. The probability of belonging to the initial group is estimated at approximately 29%, that of the second group at 40%, and that of the third at 31% (Tab 3).

**Fig 4:**
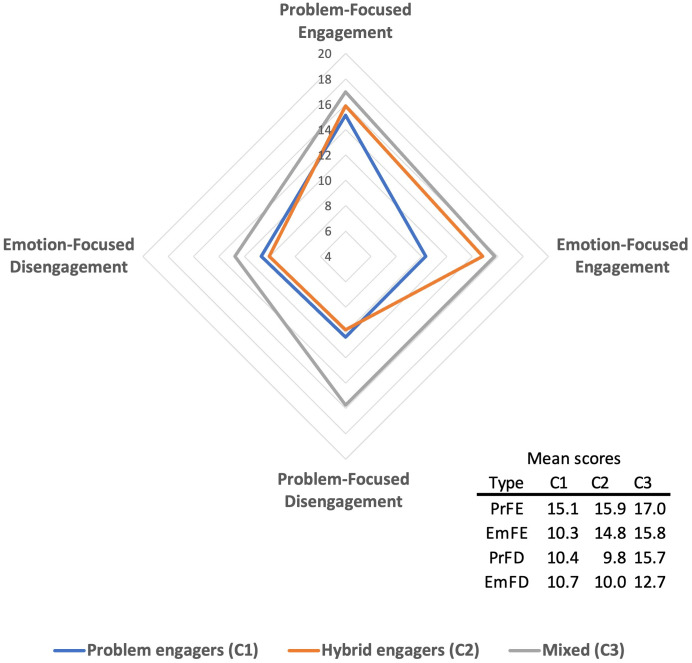
Mean CSI score for each dimension by the classes. Source: own calculations based on the 2023 MICLACAS survey.

There are significant discrepancies in the mean scores for each coping style across the distinguished classes. Class 3 exhibits the highest scores across all four dimensions. Class 1 demonstrates a notably lower utilization of emotion-focused engagement strategies, as illustrated in Fig 4.

The analysis of responses to each question revealed the coping patterns of each group, thus enabling the creation of a typology of Venezuelan migrants in Peru in terms of their coping strategies (Table 3). We label them in the following way:

- ***Problem engagers*** (class 1) are individuals who predominantly utilize problem-focused engagement strategies. It is noteworthy that this group employs only two strategies with high frequency. The probability of answering coping questions exceeded 0.75 exclusively in the case of problem-focused engagement, as evidenced by responses such as “*I look for the silver lining or try to look on the bright side of things*” and “*I tackle the problem head on*”. In contrast, the likelihood of usage of other types of coping strategies was exceedingly low. Such individuals less often apply disengagement strategies and emotion-focused strategies. The group was designated *‘problem engagers’*, despite the lowest probability of utilizing a problem-focused engagement strategy among all groups, because these individuals predominantly use this strategy.
- ***Hybrid engagers*** (class 2) also employ problem-focused engagement strategies but complement them with emotion-focused engagement ones. These individuals frequently seek for support from family and friends in discussing stressful situations (probability 0.79) and requesting assistance or guidance (0.84). In total, they used four engagement coping strategies with considerable frequency: two problem-focused and two emotion-focused.
- **Mixed strategy users** (class 3) **–** persons the combine engagement (problem and emotion-focused) with disengagement strategies trying to avoid thinking about problems (probability of 0.91), hoping for a miracle (0.78) or keeping their thoughts and feelings to themselves (0.78). These individuals demonstrate the highest utilization of coping strategies, with a total probability of high frequency usage observed for eight coping strategies (two problem-focused engagement, three emotion-focused engagement, two problem-focused disengagement and one emotion-focused disengagement).

In general, we find a very high application of problem-focused engagement strategies across all groups (see Table 4). This can be related to the migration status of the respondents. All can be positively selected from the general population of Venezuela in terms of a strong focus on active problem-solving, as the decision to migrate and its execution belong to this subgroup of coping styles. Therefore, it may be posited that this is an inherent characteristic of migrants, not solely those from Venezuela. For example, relatively high scores of problem-focused engagement strategies, like planning, positive reframing, were found among Ethiopian migrant returnees [57], Ukrainian refugee women [58] or Syrian refugees [13]. We expect that the frequency of usage of this style within the Venezuelan population will be markedly lower, particularly in light of the fact that approximately eight million individuals have departed from the country. Unfortunately, no studies on this topic have been conducted in Venezuela, according to our knowledge. The second and third groups, which we have designated as hybrid engagers and mixed strategy users, respectively, may be linked to familism, which is a typical characteristic of Latin American populations, and observed in relatively high scores on emotion-focused engagement strategies in those populations, like in Peru [59]. A strong attachment to the family network and an obligation to provide assistance in navigating the challenges and difficulties encountered by its members facilitate the utilization of emotion-focused engagement coping strategies among migrants. The very interesting finding is related to the third group which is the only one that frequently employs disengagement strategies. The responses of this group can be seen to present a contradiction. On the one hand, they frequently employ a strategy to express their feelings in order to reduce stress; on the other hand, they tend to keep their thoughts and feelings to themselves. This phenomenon is difficult to explain. We hypothesize that perhaps they are more prone to share the feelings but keep their thoughts to themselves. This aspect of our study needs a further investigation. The second disengagement strategy is wishful thinking and the belief in miracles. According to us, this can be embedded in religious beliefs and the significance of God in the lives of individuals and its influence in their everyday life shared by many Latin Americans and migrants from the region, which can be defined as religious coping [59,60].

**Table 4:** Probability of responding positively to each question of the CSI-SF instrument.

Coping	Indicator	Question	Class 1	Class 2	Class 3
			Problem engagers	Hybrid engagers	Mixed
Probability of membership			0.29	0.40	0.31
			[0.21;0.38]	[0.30;0.51]	[0.30;0.51]
Problem-Focused Engagement	PrFE_1	I make a plan of action and follow it	0.40	0.51	0.60
	PrFE_2	I look for the silver lining or try to look on the bright side of things	0.78	0.90	0.96
	PrFE_8	I tackle the problem head on	0.80	0.86	0.94
	PrFE_9	I step back from the situation and try to put things into perspective	0.42	0.47	0.66
Emotion-Focused Engagement	EmFE_5	I try to let my emotions out	0.20	0.20	0.41
	EmFE_6	I try to talk about it with a friend or family	0.22	0.79	0.78
	EmFE_11	I let my feelings out to reduce the stress	0.15	0.50	0.84
	EmFE_13	I ask a close friend or relative that I respect for help or advice	0.00	0.84	0.80
Problem-Focused Disengagement	PrFD_4	I hope the problem will take care of itself	0.10	0.14	0.41
	PrFD_7	I try to put the problem out of my mind	0.35	0.26	0.70
	PrFD_12	I hope for a miracle	0.21	0.41	0.78
	PrFD_14	I try not to think about the problem	0.16	0.18	0.91
Emotion-Focused Disengagement	EmFD_3	I try to spend time alone	0.29	0.30	0.36
	EmFD_10	I tend to blame myself	0.13	0.07	0.31
	EmFD_15	I tend to criticize myself	0.10	0.17	0.42
	EmFD_16	I keep my thoughts and feelings to myself	0.55	0.44	0.78

Source: own calculations based on the 2023 MICLACAS survey.

Note: Positive means in this context answering, “*often*” or “*almost always*”.

The LCA model was employed to predict the membership of each respondent in one of three distinct groups, with logistic regression models subsequently applied to facilitate a better understanding of their socio-demographic characteristics. The results of the models for the likelihood of belonging to each subgroup (problem engagers, hybrid engagers and mixed) indicate that individuals with a pessimistic outlook and with higher levels of education are more likely to be classified as *problem engagers* (Table 5).

**Table 5:** Main results of the logistic models; dependent variables: 1. Being problem engager, 2. Being a hybrid engager, 3. Being mixed-strategy users.

Independent Variables	Problem engagers (C1)			Hybrid engagers (C2)			Mixed (C3)		
	b		SE	b		SE	b		SE
Main Characteristics									
Sex (ref. Male)									
Female	0.147		0.251	−0.746	***	0.249	0.705	***	0.266
Age (ref. 18–29)									
30–44	0.278		−0.290	−0.277		0.280	0.422		0.302
45+	−0.566		0.370	−0.166		0.345	0.755	*	0.355
Education (ref. Primary&lower)									
Secondary	0.621		0.450	0.799	*	0.447	−1.188	***	0.394
Postsecondary+	0.773	*	0.461	1.037	**	0.457	−1.664	***	0.421
Optimism (ref. Pesimists)									
Moderate Optimism	−0.729	**	0.306	0.222		0.323	0.612	*	0.342
High Optimism	−0.821	**	0.356	0.780	**	0.360	0.013		0.399
Migration Intentions (ref. Leave Peru)									
Stay in Peru	0.026		0.265	−0.074		0.262	0.057		0.275
Constant	−0.717		0.493	−1.179	**	0.51	−0.659		0.471
Wald chi-square	13.28			21.92			33.8		
df	8			8			8		
Pseudo R-Square	0.0345			0.0548			0.0893		
N	305			305			305		

* p < 0.1; ** p < 0.05; *** p < 0.01

Source: own calculations based on the 2023 MICLACAS survey.

With respect to the subgroup of *hybrid engagers*, the probability of belonging to this subgroup is greater for males, those with higher levels of education and those with more optimistic outlook. The likelihood of employing a *mixed* set of coping strategies is greater among women, individuals aged 45 and above, those with lower levels of education and those who exhibit moderate optimism.

## Conclusions

The present study offers insights into the coping strategies employed by Venezuelan migrants. The population residing in Peru predominantly selects for engagement strategies over disengagement strategies when confronted with stressful situations. The application of latent class analysis has enabled the identification of three distinct homogeneous groups of migrants, characterized by differing patterns of coping strategy utilization on the individual level. These are designated as *problem-engagers*, *hybrid engagers* and *mixed-strategy users*. However, it must be highlighted that in all groups the highest utilized strategy was *problem-focused engagement*. The regression analysis reveals that there are significant differences in the socio-economic characteristics of these groups. The existence of hybrid engagers and mixed groups is consistent with existing literature on Latin American populations, which typically exhibits traits are related to familism and a strong interpersonal connection with family and friends, as well as a tendency to religious coping.

It is noteworthy that a subgroup, designated as ‘problem-engagers’, employ significantly less often emotional-focused strategies, instead prioritizing problem-focused engagement activities. Nevertheless, there is no notable distinction between this subgroup and other subgroups with regard to the frequency of utilization of problem-focused engagement strategies, which is a prevalent phenomenon among all Venezuelan migrants in Peru. However, there is a notable discrepancy in the frequency of application of other types of strategies. It seems that they are less inclined to seek solace in their familial and social networks as a means of coping with challenging circumstances. This may be attributed to their more pessimistic traits, as identified through the LOT-R instrument. The limitations of our study preclude a more in-depth investigation of this phenomenon. Nevertheless, this is a topic that should be the subject of further investigation.
